# Murine malaria is associated with significant hearing impairment

**DOI:** 10.1186/1475-2875-9-159

**Published:** 2010-06-11

**Authors:** Joachim Schmutzhard, Christian H Kositz, Peter Lackner, Anelia Dietmann, Marlene Fischer, Rudolf Glueckert, Markus Reindl, Kurt Stephan, Herbert Riechelmann, Annelies Schrott-Fischer, Erich Schmutzhard

**Affiliations:** 1 Department of Otorhinolaryngology, Innsbruck Medical University, Anichstraße 35, 6020 Innsbruck Austria; 2 Department of Neurology, Innsbruck Medical University, Anichstraße 35, 6020 Innsbruck Austria; 3 Department for Hearing, Speech, and Voice Disorders, Innsbruck Medical University, Anichstraße 35, 6020 Innsbruck Austria

## Abstract

**Background:**

*Plasmodium falciparum *malaria has been suspected to cause hearing loss. Developmental, cognitive and language disorders have been observed in children, surviving cerebral malaria. This prospective study aims to evaluate whether malaria influences hearing in mice.

**Methods:**

Twenty mice were included in a standardized murine cerebral malaria model. Auditory evoked brainstem responses were assessed before infection and at the peak of the illness.

**Results:**

A significant hearing impairment could be demonstrated in mice with malaria, especially the cerebral form. The control group did not show any alterations. No therapy was used.

**Conclusion:**

This suggests that malaria itself leads to a hearing impairment in mice.

## Background

With more than 247 million cases in 2006 malaria is one of the most frequent infectious diseases world wide[1]. The most severe course of the human disease is caused by *Plasmodium falciparum*, leading to multi-organ disease. In particular cerebral malaria (CM) is potentially leading to a wide range of neurocognitive sequelae[2]. Furthermore, it has been shown that severe falciparum malaria may lead to an acquired language disorder[2].

Language development in childhood needs an intact and perfectly functioning hearing system - from the outer ear canal to the sensory cerebral cortex. So far, in malaria research no dedicated and specific attention has been paid to the involvement of the inner ear. Only very few authors have reported an acquired hearing impairment possibly caused by falciparum malaria[3,4]. To shed light into this aspect, a cerebral malaria experiment in *Plasmodium berghei *ANKA infected C57BL/6J mice - a well defined model for severe/cerebral malaria - was performed evaluating the hearing threashold with auditory evoked brain stem responses (ABRs) before infection and at the peak of the disease[5,6].

The primary aim of this study was to evaluate a possible change of the hearing threshold in mice with CM and malaria without cerebral involvement.

## Methods

### Study design

The animal study conformed to the Austrian guidelines for the care and use of laboratory animals and were approved by the Austrian Ministry for Education, Science and Culture with the reference number do. Zl. A08/4102. Three different animal groups were studied. One group contains the mice suffering from cerebral malaria (CM). The second group contains mice, which are infected with malaria, show a parasitaemia comparable to the cerebral malaria mice, but did not develop a cerebral involvement (non-CM). The third group were healthy non-infected animals, which were kept at equal housing, environmental and experimental conditions.

Prior to infection a baseline hearing test with ABRs was performed for the frequencies 8 kHz, 13 kHz, 36 kHz and a click sound. Only animals with clearly evokable and readable initial ABRs were included into the study and infected on the same day.

To monitor the course of the disease the mice were subjected to a daily evaluation of the SHIRPA score. The CM group is expected to develop signs and symptoms between day 5 and 11 after infection. At the peak of the cerebral disease a second ABR measurement was performed and thereafter the animals were sacrificed. Animals surviving day 10 do not develop CM anymore [6]. These animals were used as the non-CM control group and, the second ABR measurement was done on the 11^th ^day immediately before sacrification.

The results of the ABR were compared between groups and to the baseline data.

After the ABR measurements the CM mice were sacrificed and their brains examined for vascular affection.

### Auditory brainstem responses

The auditory brain stem responses were measured with ABR machine provided by ZLE - Systemtechnik^©^, Munich Germany. Three frequencies 8 kHz, 13 kHz, 36 kHz and a click sound were measured to evaluate the hearing threshold. The right ear of each mouse was examined. Subsequently the ABRs were measured in an electrically shielded sound attenuating chamber. The potentials are gained by means of three subcutaneous needle-electrodes. The positive electrode is placed at the vertex, the negative electrode at the bulla and the ground electrode at the ipsilateral leg. Tones of 8, 13, 36 kHz and a click sound are used as stimuli. The clicks are produced by two different transducers. Starting with 80 dB the stimuli are decreased by 10 dB. The ABR thresholds are determined as the minimum stimulation level, that produces a clearly recognisable potential. The signals are amplified by a physiologic amplifier and filtered. The responses of 500 stimuli are averaged by means of computerized data acquisition synchronized to stimulus onset. The resulting wave responses were interpreted in a blinded manner by two independent ENT-specialists (JS, ASF).

The anesthesia is performed with an Ohmeda Isotec 5 Vaporisator (Duisburg, Germany). With a continuous flow of 3 l/minute oxygen 1% isoflourane is vaporized. The nose and mouth of the mouse are loosly fitted into the dispenser mask. The excess anaestetic and air is disposed of by wide loomed noiseless suction.

### Animal model

Six to eight week old C57BL/6J mice (Charles River Laboratories, Sulzfeld, Germany) are used as cerebral malaria susceptible strains. The mice are housed under standard conditions in the animal housing facility of the Medical University Innsbruck, Austria. After an accommodation period for approximately three to five days, the animals are taken for the study. The C57BL/6J mouse is a strain susceptible to blood stages of *P. berghei *ANKA. After the determination of the ABR thresholds as described above, the mice are infected with an intraperitoneal application of 1 × 10^6 ^parasitized erythrocytes of a homologous donor, which had been infected with frozen polyclonal stocks of *P. berghei *[7]. The parasitaemia was monitored by daily blood smears of tail blood. The smears are stained by Wright stain.

The progress of the disease was evaluated with the SHIRPA score as described by Lackner *et al *[6]. The SHIRPA score is a behavioural screening battery consisting of 40 simple tests, which are arranged in the five subgroups: reflex and sensory function, neuropsychiatric state, motor behaviour, autonomous function, muscle tone and strength. A total SHIRPA score below 15 is considered as severe cerebral malaria, between 15 and 25 as moderate and above 25 as mild [8]. When typical signs and symptoms of cerebral malaria, like convulsions, paralysis and coma appear, the mice are considered at the peak of the disease.

### Histologic workup

After the ABR measurement the deeply anaesthesized animals were sacrificed by an intraperitoneal thiopental overdose for futher histological workup. The fixation and histologic work-up was done according to a standard procedure as described by Lackner [6].

### Statistical evaluation

The statistical work up was done with SPSS (v.17.0, Chicago, USA). The baseline measurement and the second measurement was compared with the Wilcoxon signed rank sum test. This procedure was done for each group separately. The graphical presentation of the data was done with Graphpad Prism 5.01 (Graphpad Softwareincorporation, La Jolla, USA).

## Results

Thirty C57BL/6J mice were included into the study. After baseline measurement 29 mice were included into the study. One mouse showed - at the initial measurement - a pathological hearing threshold and was excluded.

Twenty mice were infected as described above and all developed similar parasitaemia. Out of these 20 mice ten developed cerebral malaria (CM) and 10 did not develop a cerebral manifestation (non-CM).

The CM group showed an average SHIRPA score of 28.7 at the start of the study, which decreased to an average of 16.5 points. Three of the mice developed a severe CM with a SHIRPA score below 15. With a scoring between 15.9 and 21.6 the other mice could be judged as moderate CM. No animal was in the mild CM group.

The non CM group scored an average of 28.2 SHIRPA points at the beginning of the study and decreased to an average of 25.4 at the timepoint of the second measurement. Five of the the non CM mice showed a mild temporary drop in the SHIRPA score, stabilizing at a lower level. The healthy control group was not retested, all mice remained in a stable condition.

**Figure 1:**
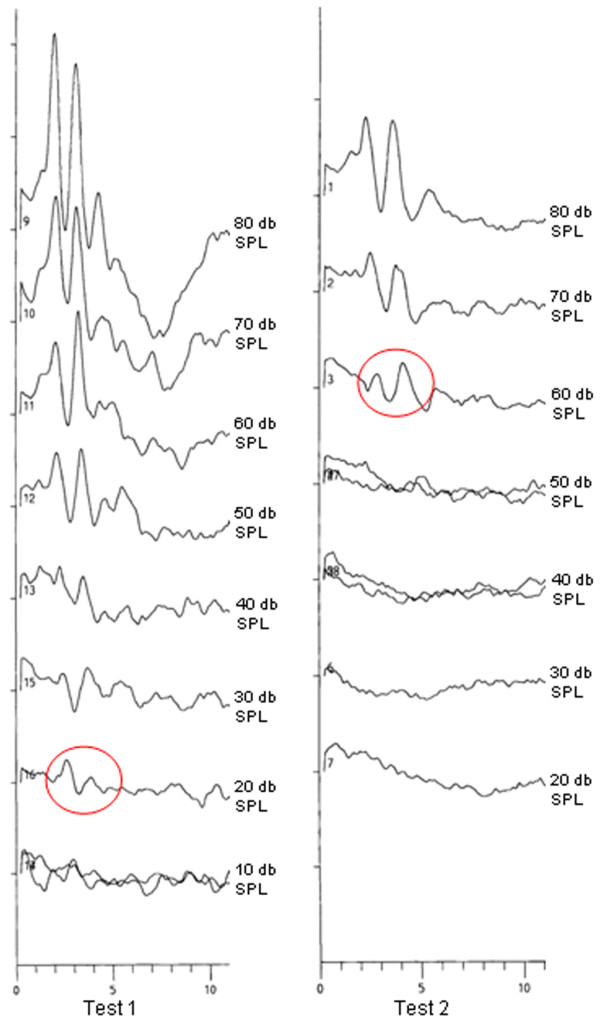
**shows an example of the ABR findings at the frequency of 13 kHz in a cerebral malaria mouse**. Test 1 represents the baseline measurement. Test 2 is the ABR finding at the peak of the cerebral malaria. The red circles indicate the last positive counted wave. Therefore a hearing threshold of 20 dB SPL is counted in test 1 and a threshold of 60 dB in test 2. (SPL:Sound Pressure Level)

Six mice of the CM group developed various degrees of hearing impairment with a maximum loss of up to 40 dB in various frequencies. (Figure 1, 2a-d) The animals with the most severe hearing impairment were categorized as severe CM with a Shirpa scoring under 15. The statistical analysis of the CM group revealed a significant alteration of the hearing threshold in the frequences 8 kHz (p = 0.047) and 36 kHz (p = 0.023), a trend could be demonstrated for 13 kHz (p = 0.059). No change was found for the click sound.

**Figure 2:**
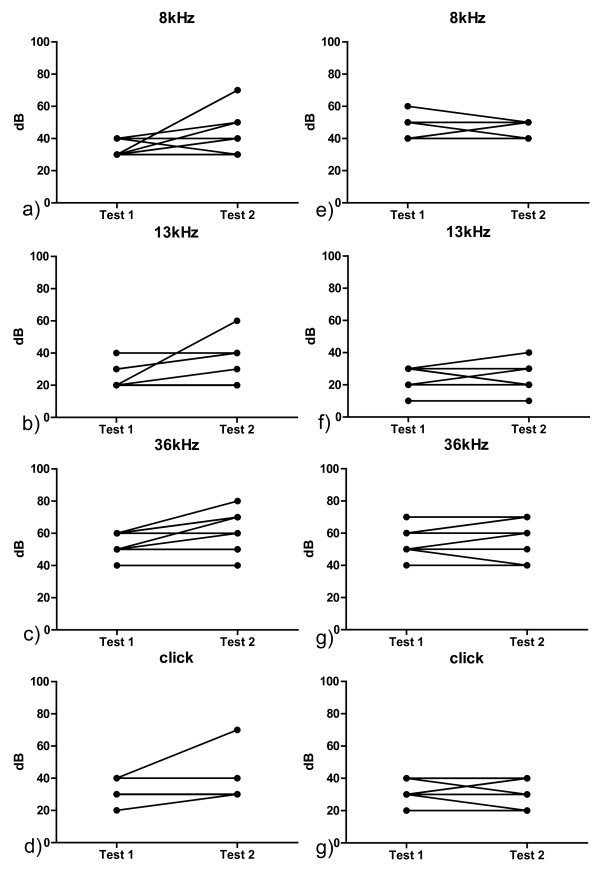
**shows the audiologic results of the CM group and the healthy control group**. a-d) represent the CM group. Test 1 is done at baseline screening. Test two at the peak of the clinical disease. e-f) list the findings of the control group. Test 1 is done at baseline screening. Test two at the 11^th ^day after the primary testing.

The non-CM group consisted of 10 mice. Seven of these animals developed a hearing loss up to 20 dB in the various frequencies comparing the baseline and the measurement on day 11. All five non CM mice with a recorded temporary drop of the SHIRPA score showed this hearing impairment. The statistical evaluation of the non-CM group showed a significant hearing impairment at frequency 8 kHz (p = 0.014) and a trend at the frequency 13 kHz (p = 0.059). No significant alteration was marked for 36 kHz and Click.

Nine mice served as control group. At both timepoints of measurement no significant hearing alterations could be detected in all evaluated frequences (Figure 2e-f). Six times a decrease of 10 dB could be noted and six times an increase of 10 dB in all measured frequences.

**Figure 3:**
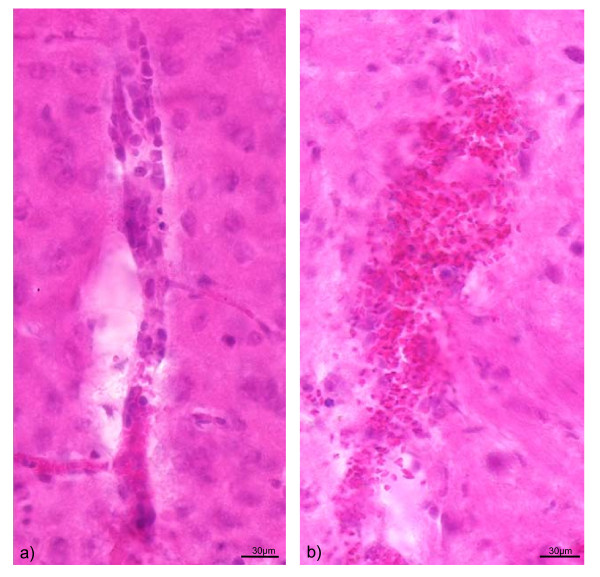
**shows the histology of the brain of a mouse with cerebral malaria and hearing impairment**. The typical signs of leucocyte sequestration (**a**) and microhaemorrhages (**b**) are shown. (Hematoxilin Eosin staining; magnification 400 ×)

The histological examination of all ten CM mice showed the expected vascular affection with parenchymal microhaemorrhages and leukocyte sequestration (Figure 3) confirming the cerebral manifestation.

## Discussion

The existence of a malaria related hearing loss has been suspected in African CM children without quantifying the burden of the problem nor addressing the pathophysiological basis[3,4]. This study demonstrates in a prospective way, that malaria leads to a significant hearing impairment in mice. When comparing the results of the different subgroups an affection at all measured frequencies could been shown in the CM group, but only at the lower and middle frequencies in the non-CM group, as expected the control did not show any significant hearing alterations.

The cerebral affection of the measured animals was confirmed by histology.

Zheng *et al *[9] described the hearing threshold for the C57BL/6J mouse on a similar level as found in the baseline ABR proving the quality of the the experimental set-up. The anesthesia used in Zhengs study was an intraperitoneal injection of ketamine and xylazine. Most studies evaluating ABR use an intraperitoneal injection to avoid a possible negative influence of isofluorane on the ABR results. Isoflourane has been described to lead to a significant latency increasement, but does not influence the amplitude in rats[10]. Aware of these possible side effects, the isofluorane anesthesia was chosen, which can be controlled much easier, to avoid an accidental early death of these very sick animals at the timepoint of the second ABR testing.

The pathophysiological processes leading to these findings are unclear. The ABR results show an equal decrease of the amplitudes in all measured peaks. A pathologic alteration of the brain stem and the upper audiologic centers would result in a decrease of the amplitude of peak three to five, whereas the peak one and two should not be affected [11]. Therefore a pathologic alteration of the brain stem is highly unlikely

Malaria retinopathy has been suggested to be the result of adherence and sequestration of *P. falciparum*-infected erythrocytes in the microvascular system leading to impaired microcirculation [12]. The anatomical situation of the ocular and the inner ear perfusion is similar. Both the ophthalmic artery and the labyrinth artery originate from cerebral vessels. Thus, it seems conceivable that impairment of cerebral microcirculation within the central nervous system is very likely to include both organs [8]. The spiral modiular artery enters the cochlea at the basal turn and ends at the apical turn [13]. Therefore, disturbances of the microvascular system and impairment of perfusion of the cochlea should effect the apical turns more than the basal. With a significant hearingloss at 8 kHz - a frequency which is located more apically in the cochlea -the hypothesis of a microvasvular affection in the CM group is supported. The non-CM group with a significant impairment of the lower and middle frequencies suggests an equivalent interpretation possibility.

The majority of the animals (five of seven) with hearing impairment did show a mild temporary drop in the SHIRPA score, suggesting a mild cerebral/vascular affection as described by Cabrales et al.[6,14]. This circumstance further supports the hypothesis that the vascular pathophysiology in cerebral malaria leads to an affection of the inner ear. To clarify this pathophysiological concept additional studies in other non-CM models are required.

The affection at 36 kHz, a frequency which is located at a more basal turn of the cochlea, and the minor affection of the more apically positioned 13 kHz is hardly explainable by this interpretation. Other pathologic alterations are known to affect the inner ear with various inflammatory mediators [15]. A similar effect onto the inner ear could be possible since it has been shown that a wide variety of inflammatory mediators are released during the course of CM or severe malaria, respectively. For example, an increase of the soluble Endoglin has been shown in severe malaria [16]. Endoglin is a co-receptor of the TGF- beta system [16]. Satoh *et al *showed an involvement of this TGF-beta system in the cochlea immune response [17] rendering this hypothesis - that various inflammatory mediators of malaria also affect the inner ear.

Furthermore, *P. falciparum *malaria is known to cause language disorders in children [2]. This clinical finding has been suggested to be a consequence of cerebral injury. Carter *et al *[2], when discussing these findings, did not list any audiologic testing nor any hearing thresholds. In early childhood, i.e. during the period of language development, hearing impairment is known to cause a retardation or, even, impairment of the language development [18].

Summarizing, malaria, especially the cerebral form, has been shown to cause hearing impairment in the murine malaria model. Further studies are nescessary to understand the pathophysiological alterations in the inner ear and to evaluate the impact of these findings for humans.

## List of abbreviations

ABRs: auditory evoked brainstemresponses; CM: Cerebral Malaria; non-CM: non Cerebral Malaria; kHz: kilo Hertz.

## Competing interests

The authors declare that they have no competing interests.

## Authors' contributions

JS: has drafted the manuscript and evaluated the ABR data.

CK AD MF RG MR: have made substantial contribution in the concept and the coordination of the animal experiment and meassurement as well as in the acquisation of the data.

KS: has supervised the ABR meassurement and was essentially involved in the establishment of the ABR meassurement facillity

ASF: evaluated the ABR data and critically revised the manuscript.

HR: have given final approval of the version to be published

PL: has done the statistical calculation

ES: made the concept and the design of the study

All authors read and approved the final manuscript.
